# MINFLUX nanometer-scale 3D imaging and microsecond-range tracking on a common fluorescence microscope

**DOI:** 10.1038/s41467-021-21652-z

**Published:** 2021-03-05

**Authors:** Roman Schmidt, Tobias Weihs, Christian A. Wurm, Isabelle Jansen, Jasmin Rehman, Steffen J. Sahl, Stefan W. Hell

**Affiliations:** 1 Abberior Instruments GmbH, Göttingen, Germany; 2 Abberior GmbH, Göttingen, Germany; 3 Department of NanoBiophotonics, Max Planck Institute for Biophysical Chemistry, Göttingen, Germany; 4 Department of Optical Nanoscopy, Max Planck Institute for Medical Research, Heidelberg, Germany

**Keywords:** Fluorescence imaging, Super-resolution microscopy

## Abstract

The recently introduced minimal photon fluxes (MINFLUX) concept pushed the resolution of fluorescence microscopy to molecular dimensions. Initial demonstrations relied on custom made, specialized microscopes, raising the question of the method’s general availability. Here, we show that MINFLUX implemented with a standard microscope stand can attain 1–3 nm resolution in three dimensions, rendering fluorescence microscopy with molecule-scale resolution widely applicable. Advances, such as synchronized electro-optical and galvanometric beam steering and a stabilization that locks the sample position to sub-nanometer precision with respect to the stand, ensure nanometer-precise and accurate real-time localization of individually activated fluorophores. In our MINFLUX imaging of cell- and neurobiological samples, ~800 detected photons suffice to attain a localization precision of 2.2 nm, whereas ~2500 photons yield precisions <1 nm (standard deviation). We further demonstrate 3D imaging with localization precision of ~2.4 nm in the focal plane and ~1.9 nm along the optic axis. Localizing with a precision of <20 nm within ~100 µs, we establish this spatio-temporal resolution in single fluorophore tracking and apply it to the diffusion of single labeled lipids in lipid-bilayer model membranes.

## Abstract (web summary)

Minimal photon fluxes (MINFLUX) has enabled molecule-scale resolution in fluorescence microscopy but this had not been shown in standard, broadly applicable microscopy platforms. Here the authors report a solution to allow normal fluorescence microscopy while also providing 1-3 nm 3D resolution.

## Introduction

Fluorescence microscopy recently experienced a second resolution boost due to the synergistic combination of the specific strengths of the coordinate-targeted super-resolution^1^ family represented by STED^2–4^ and RESOLFT^5–7^ and its coordinate-stochastic counterpart comprising PALM/STORM^8–10^ and PAINT^11^. The resultant concept called MINimal photon FLUXes (MINFLUX)^12–14^ closed the prevalent resolution gap from ~20 to 30 nm in STED, PALM/STORM and other fluorescence nanoscopies^15–17^ to the 1–5 nm size scale of the molecules themselves.

MINFLUX nanoscopy separates the fluorophores in the sample by activating and deactivating them individually per diffraction region, whereas the identification of the fluorophore coordinate is based on the important rationale of “injecting” a reference coordinate in the sample using a structured optical beam, such as a donut with a central intensity minimum (zero). The position of the zero in the sample defines the targeted sample coordinate. Note that the MINFLUX concept^12,18,19^ equally applies to entire sets of reference coordinates (line- and point-like zeros) and parallelized (camera) detection in the widefield. Coordinate targeting enables a well-controlled and therefore photon-efficient localization of fluorescent molecules, because the fluorophore coordinate to be determined is no longer “passively” found by establishing the center of a feeblefluorescence diffraction spot emerging on a pixelated detector such as a camera. Instead, the fluorophore is localized by actively targeting the zero of the excitation donut to the fluorophore. Concretely, the excitation intensity zero is brought as closely as possible to the molecule in well thought-out iterations, until the detected fluorescence rate approximately matches that of the background noise. In this closest proximity, only a minimal number of fluorescence photons are needed to gain maximal localization precision, because establishing the remaining distance between the coordinate targeted by the donut zero and the molecular position requires much fewer detected photons. Thus, “injecting” or targeting a reference coordinate in the sample shifts the burden of requiring many fluorescence photons for localization to the inexhaustible number of photons in the donut-shaped excitation beam. (Compare the original *gedankenexperiment* of the demon to illustrate the MINFLUX concept^12^).

Since MINFLUX localization is no longer limited by waiting for large numbers of fluorescence photons, this nanometer-precise localization is substantially faster^13^ than the “passive” camera-based localization used in PALM/STORM. Note that the idea of optically injecting a coordinate using a donut zero is present in the original STED concept. For STED microscopy it is evident that, in the absence of background, a single detected photon suffices to prove the presence of a fluorophore right at the coordinate targeted by the donut zero. There as well, the emitting fluorophore is perfectly localized by the photons injected by the STED beam^20^.

It has been shown^12^ that the minimum localization precision achievable with an unbiased estimator, i.e. the Cramér-Rao lower bound (CRLB) for localization of a fluorophore located within the region of diameter *L* outlined by the targeted coordinate pattern (TCP), is given by $\sigma \ge \frac{L}{{\gamma\sqrt N }}$, with *N* denoting the sum of photons detected with the zero placed at the coordinates of the TCP; γ is a constant that depends on the TCP’s geometry. While the dependence on the TCP diameter *L* is linear, the dependence on the number of detected photons *N* merely follows the well-known inverse square root relation. Therefore, bringing the excitation donut zero closer to the fluorophore position, i.e., a controlled reduction of the TCP diameter *L*, increases the localization precision more effectively than waiting for larger numbers *N* of detected photons. This fundamental fact is at the heart of the MINFLUX iteration, which plays out the central idea of bringing the donut zero virtually to spatial coincidence with the probed fluorophore, a procedure only limited by background noise. For the general case of a TCP comprising a set of outer triangular plus central probing points^14^, a successive zooming-in on the molecule with stepwise-reduced $L_k$ (with $L_k$ chosen to be three times $\sigma _{k - 1}$, i.e., the uncertainty in the previous iteration) is a workable strategy for refining the position estimate. After $k$ iterations, and therefore for $N_{t} = k \times N$ of detected photons in the case of *N* photon counts per iteration, the CRLB becomes^14^: $\sigma _k \ge \frac{{L_k}}{{\gamma\sqrt N }} = \frac{{3 \times \sigma _{k - 1}}}{{\gamma\sqrt N }} = \frac{{3 \times L_{k - 1}}}{{\left( {\gamma\sqrt N } \right)^2}} = \cdots = \frac{{3^{k - 1}}}{{\left( {\gamma\sqrt N } \right)^k}}L_1 \propto k^{\frac{k}{2}}\frac{{L_1}}{{N_{t}^{\frac{k}{2}}}} \cdot$ Already four steps (*k* = 4), as demonstrated, yield $\sigma _4 \propto 1/N_{t}^2$, i.e., an inverse quadratic as opposed to an inverse square root dependence on the number of detected photons. More iterations readily yield an even higher order reflecting an exponential relationship. Crucially as well, the *N* collected in each iteration need not be identical. Rather, the photon numbers may be individually adjusted for their most efficient expenditure in the iterative procedure.

As the three-dimensional (3D) localization precision attains standard deviations of 1–3 nm, new challenges arise, in particular with respect to vibrational isolation and adaptability to new imaging situations. As a matter of fact, it has not been clear whether these challenges can be met in regular microscopy settings and MINFLUX nanoscopy turned into a super-resolution method that is broadly applicable. We here describe a solution that maintains normal fluorescence microscopy operations while concomitantly enabling molecule-scale 3D resolution. Our results—with demonstrated resolution on par with that from the most recent report of cellular MINFLUX nanoscopy^14^—clearly show that MINFLUX nanoscopy and single-molecule tracking is transferrable to widely usable microscopy platforms, thus opening up windows of investigation in the life sciences. Moreover, our lipid molecule tracking data sets a spatiotemporal benchmark in single-molecule tracking.

## Results

### Active-feedback position stabilization enables routine MINFLUX nanometric resolution on a standard microscope stand

**Fig. 1:**
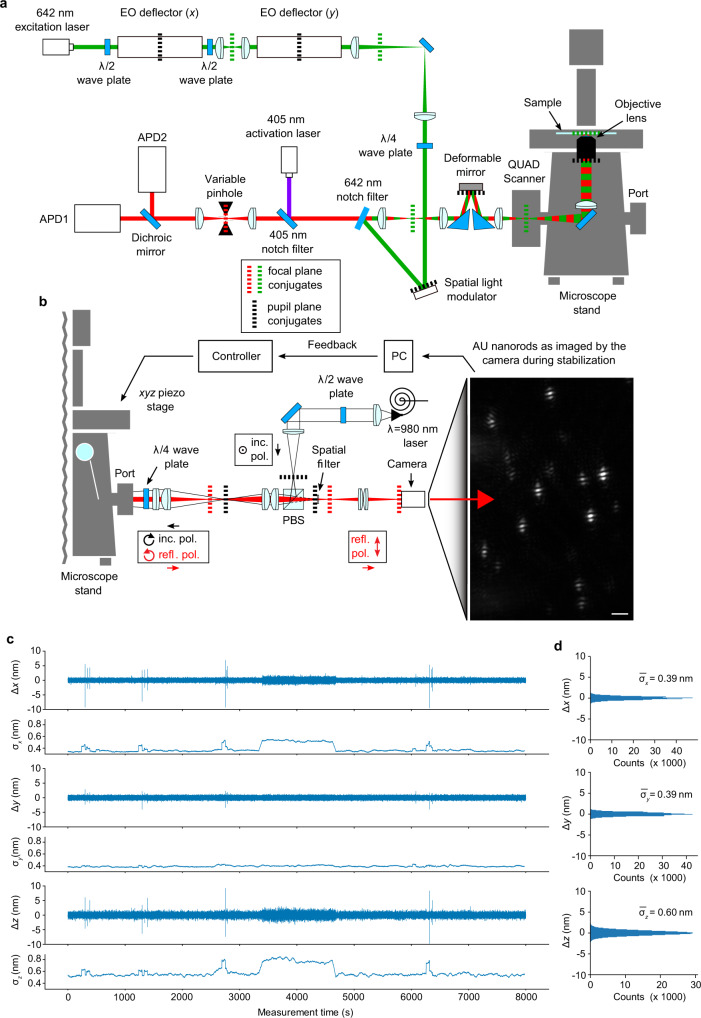
MINFLUX fluorescence nanoscope with optical-feedback stabilization on an all-purpose microscope stand: sub-nanometer stability. **a** Optical arrangement. An excitation beam (shown in green) is electro-optically deflected in *x*,*y*, spatially phase-modulated for conversion into a donut-shape, overlapped with an activation beam (purple) and, after passing a deformable mirror and a galvanometer scanner in a 3D scanning assembly, focused into the sample on top of an all-purpose inverted microscope stand. Fluorescence from the sample (red) is descanned by the scanning assembly and passed to a variable confocal pinhole for detection using two avalanche photodiodes (APD). A stabilization unit based on both near-infrared scattering from fiducial markers and active-feedback correction provides sub-nanometer stability. **b** Layout of the stabilization unit including an example widefield image of the scattered reflection signal of gold nanorods in the sample. **c** Sample displacements *∆x*,*y*,*z* from the target position as measured by the active stabilization over 2.2 h, and their running standard deviations *σ*_*x*,*y*,*z*_ over a 1-min window. While varying vibrational interference from the surroundings is reflected in the stabilization performance, *σ*_*x*,*y*,*z*_ typically stay well below 1 nm. **d** Histograms of the displacements and their standard deviations over the full 2.2 h interval shown in (**c**). Source data are provided as a Source Data file. Scale bar: 2 µm (**b**).

We reasoned that realizing MINFLUX on top of a typical inverted microscope platform (Fig. 1a) facilitates the combination of practicability with ultimate resolution. Microscopy workflows usually entail prior steps before a nanometer resolution image is recorded, in particular the selection of regions of interest (ROIs). The option to switch between lenses of different magnifications and complementary imaging methods, such as camera- or eyepiece-based epifluorescence, transmission, or DIC contrast, is invaluable and enables the investigation of complex biological samples. Therefore, we expended considerable effort to finding an approach for a robust stage stabilization in a closed feedback loop, so as to allow both flexible previews of large ROIs and precise measurements in desired regions applying the MINFLUX scheme.

To this end, we developed a beam scanning scheme with galvanometer and electro-optical (EO) beam deflectors operating in concert. Two pairs of galvanometer scanners implemented in a custom QUAD-Scanner design^21^ achieve a state-of-the-art *xy*-scan range, e.g. 80 × 80 µm with a ×100 objective lens or 200 × 200 µm with a ×40 objective lens, that allows to conveniently identify ROIs. In the hierarchically designed scan unit (Fig. 1a) the galvanometer, which is attached to the camera port of the microscope stand, serves as the coarser basis for additional finer and much faster electro-optic *x*- and *y*-displacements^22^. The EO *x*- and *y*-scanners are implemented serially in the laser line followed by a spatial light modulator (SLM), which introduces a helical phase shift onto the laser beam, shaping it to a donut in the focal plane. Jointly, both the galvanometer and the EO units are used to position the excitation beams in the microscope, either as a normally focused beam for confocal scanning, or as a donut with a central zero-point intensity for the MINFLUX protocol. To enable 3D MINFLUX localization, we employ a deformable mirror (DM). Appropriate alteration of the DM curvature, i.e. adding defocus phase terms, shifts the focal zero-intensity point along the *z*-axis. For 2D experiments, the DM is set to a neutral (flat-field) configuration. Fluorophore activation is accomplished within a ~400 nm diameter region in the sample using a regularly focused 405-nm beam co-aligned to the excitation beam; it is provided to the microscope without passing the EO devices.

The active sample stabilization (Fig. 1b, c and Supplementary Fig. S1) utilizes back-scattered light from reflective fiducials (e.g., gold (Au) nanorods) that are immobilized along with the biological sample (Fig. 1b). Concretely, light from a 980 nm laser source is transferred to the sample as widefield illumination by focusing the polarization component reflected off a polarizing beam splitter cube (PBS) through a quarter-wave plate retarder (λ/4) into the back focal plane (BFP) of the objective lens. Light reflected from the sample and entering the detection arm of the stabilization is consequently orthogonally polarized with respect to the incident light and thus transmitted by the PBS. To boost the relative strength of high spatial frequencies in the CCD camera image that is monitoring the sample position, we installed a spatial filter that blocks the light near the optic axis in a Fourier-conjugated plane with respect to the CCD camera sensor. As an added benefit, this configuration also blocks light that is back reflected from the objective lens. In a closed control loop, positional changes of the sample are constantly measured at a rate exceeding 40 Hz. We cross-correlate the live camera image with a prerecorded reference (*z*-) stack of images from above and below the axial setpoint and calculate the current *xy*- and *z*-position as the interpolated point of maximum similarity between the shifted image and the reference stack. Any deviation from the setpoint is immediately counteracted by a piezo-driven (*xyz*-) sample stage.

Image correlation analysis using the combined signal of multiple fiducials bears two major advantages over the tracking of a single particle. First, a higher number of detected photons per frame allows for a faster and/or more precise stabilization. Second, routine operation is facilitated as it requires only minimal effort to find a suitable imaging region, where a few gold particles fall within the 25 × 25 µm field of view of the camera. Figure 1c, d shows actual displacement data recorded during a >2-h long stability monitoring measurement. For an imaging experiment under normal laboratory environment conditions, the achieved stability is remarkable, routinely exhibiting standard deviations substantially below 1 nm over minutes and indeed up to hours if agitation of the microscope by external sources such as rapid walking or door closing are kept at a reasonable level. At these timescales, other sources of image drift that are not covered by the sample stabilization, such as overall beam line stability, may affect the measured data depending on the environmental conditions under which the microscope is operated. For the presented data, however, neither measures for tight control of temperature nor airflow had to be exerted apart from moderately sealing the microscope itself.

### Prelocalization steps for an enhanced field of view

**Fig. 2:**
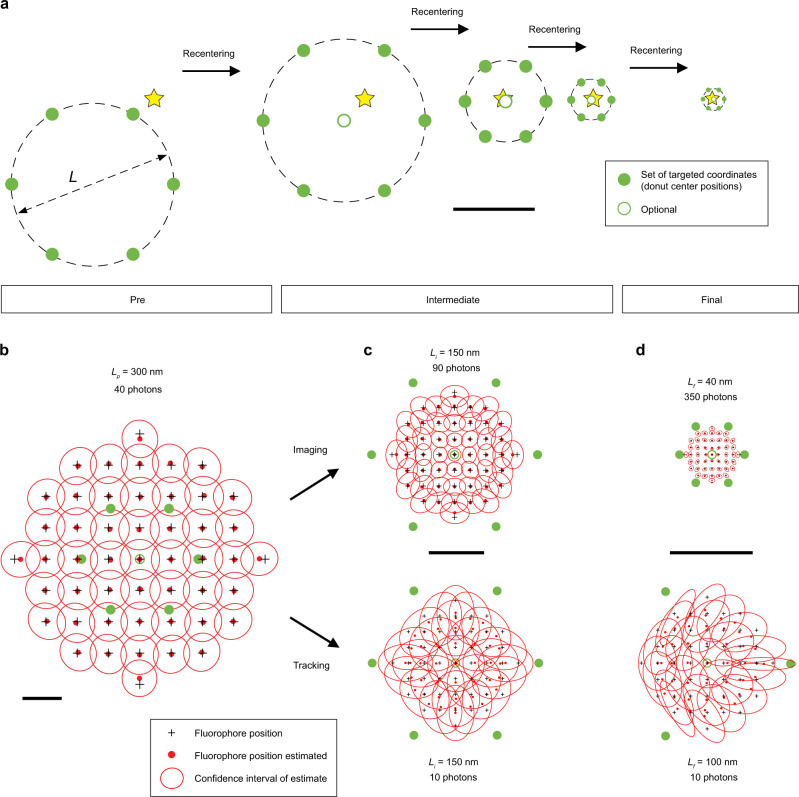
Targeted coordinate patterns (TCP) in sequential iterations for imaging and fast single-molecule tracking. **a** The TCP defines a set of relative coordinates that are used to probe the position of the fluorescent molecule (star) in the sample. In a typical 2D MINFLUX iteration, the central zero of the donut-shaped excitation beam is targeted to these coordinates that are arranged in a hexagon with diameter *L* plus an optional midpoint (all in green). After each iteration, the TCP is re-centered based on the prior estimate of the molecular position. A MINFLUX sequence defines the parameters of these consecutive localization iterations, starting with a pre-scan, followed by intermediate and final iterations which are processed during individual localization events. Scale bar: 150 nm. **b**–**d** Typical parameters and estimator performances for imaging and tracking applications. While imaging sequences tend to spend more photons on later iterations to maximize precision, tracking sequences are optimized for speed and thus spend only minimal photons on the final iteration, which tracks the molecule (**d**). Simulated molecules at positions (crosses) within the FOV and the distributions (red) of their respective estimate represented by their mean (dot) and confidence intervals (ellipsoids). Note the nonvanishing residual bias at the outmost grid locations which marks the edge of the usable field of view. In the tracking parts of (**c**, **d**), every second confidence interval is shown for clarity of display. Source data are provided as a Source Data file. Scale bars: 100 nm (**b**), 50 nm (**c**, **d**).

In practice, the initial MINFLUX localization range *L* is about the wavelength of the excitation light. To scan extended samples in a micrometer-sized ROI, our microscope spans the ROI with a hexagonal grid of scanning positions, with a mutual distance of between 10 and 50% of the excitation wavelength used. These positions are repeatedly probed during a measurement and serve as starting points for the activation, search, and localization (Fig. 2a) of fluorescent markers.

Following up to two lower precision prelocalization steps in which the galvanometric and EO scanners act in concert with an effective TCP diameter *L*_*p*_ = 300 nm and *N*_*p*_ = 40 photons (Fig. 2b), the iterative procedure (sequence) proceeds with subsequent iterations *k* which quickly zoom in on the emitter (Fig. 2c, d).

### Localization with a hexagonal-targeted coordinate pattern for iterative MINFLUX

For each localization, we targeted the excitation donut zero to the TCP, probing at the TCP multiple times during each iteration until the actual number of collected photons surpassed the preset limit *N*_*k*_. The diameter *L* of the circle encompassing the TCP is a measure of how well the zooming-in on the fluorophore position has progressed. In each iteration, we calculated the anticipated fluorophore position. In the subsequent iteration, the TCP was centered on the new position and the diameter *L* decreased according to the updated precision estimate, thus bringing the donut zero successively closer to the fluorophore. To compensate for the associated reduction in excitation intensity and to maintain the fluorescence flux, we increased the excitation power in each step accordingly. A field-programmable gate array board performed the iterative localization after identifying single active fluorophores through emission trace segmentation. Nonspecific fluorescence and other background contributions necessitate a compensatory procedure to render reliable, unbiased localization estimates without systematic errors^12,14^. We implemented such a background compensation, performing it in real-time instead of by data post-processing^12,14^.

In our experiments, we selected a TCP consisting of a hexagon with an optional central seventh position (hexagon plus center) because the resulting increase in angular uniformity of the estimator bias over the reported triangular TCP (Supplementary Fig. S2) allowed us to implement unbiasing as a simplified, one-dimensional (radial) problem that is well suited for real-time computation. The hexagonal TCP was applied in all iterations except those where we aimed for highest localization rates and avoided the additional overhead (Fig. 2d). Additional checks on the localizations comparing emission frequencies measured with the focal excitation intensity distribution being centered on the circumference of TCP circle and at its origin (center frequency ratio, CFR, see “Methods”) may serve as an additional test on whether a single fluorophore or a conglomerate is actively emitting in the field of view. In our experiments, a precision of ~2 nm is uniformly afforded within a final TCP of *L*_*f*_ = 40 nm diameter using only 350 photons (Fig. 2d). In total, a mere ~800 photons have typically been expended up to this point, and subsequently emitted photons may be used to refine the position estimate. Precisions of 1 nm and below are quickly reached for 1800–2500 photons collected in total.

### MINFLUX fluorescence imaging in cells at down to 1 nm resolution

For imaging with Alexa Fluor 647^10^, we transiently switched on a single fluorophore within the ~400 nm diameter activation region, localized it by iterative MINFLUX, and finally let it return to a long-lasting off-state. The same procedure was applied to the next fluorophore until sufficient or representative numbers of fluorophores in the structure were registered.

**Fig. 3:**
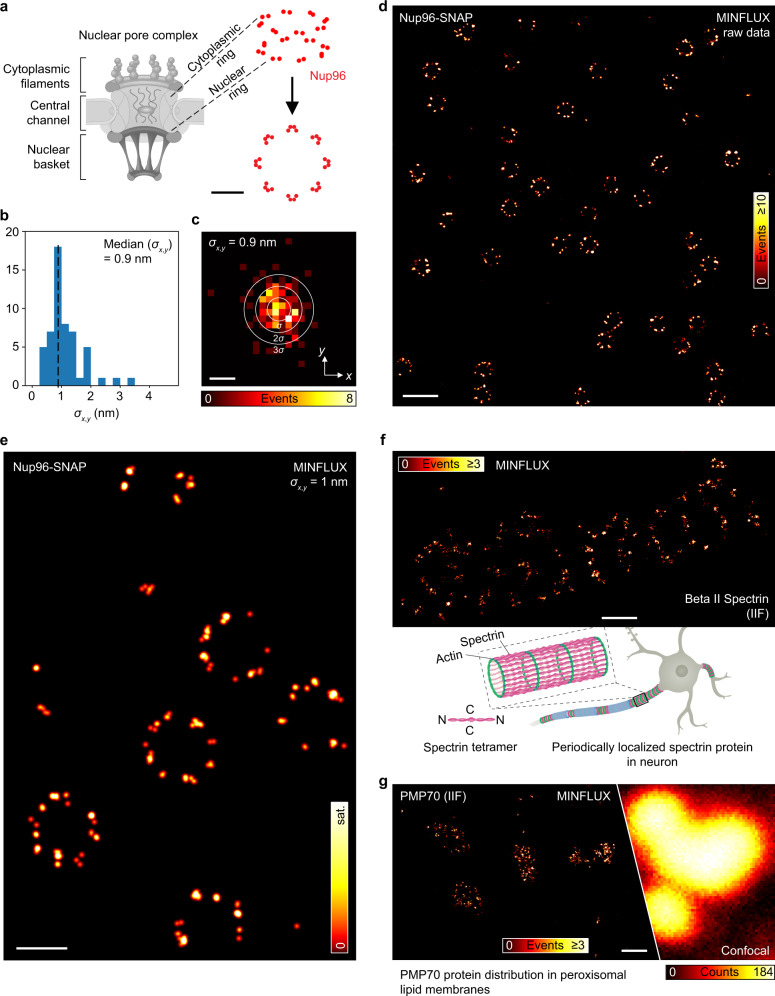
MINFLUX fluorescence imaging of labeled cellular ultrastructure down to 1 nm (standard deviation) in fluorophore precision. **a** Nup96 localizes in two eightfold-symmetric rings within the nuclear pore complex. **b** Histogram of the standard deviation from sets of sequential localizations based on 2100 photons each taken from the uninterrupted photon emission traces at the final MINFLUX iteration step (Nup96 data shown in (**d**)). Molecules providing ≥ 4 localizations per trace were considered. **c** Histogram of the distance of a localization to the mean position of a single fluorophore (data as in (**b**)). The ellipses are displayed with semi-axes of *σ*, 2*σ*, and 3*σ* in length, with *σ* the precision obtained from a combined analysis of the statistical localization spread (standard deviation) in *x* and *y*. **d** MINFLUX nanoscopy reconstruction of Nup96-SNAP labeled with Alexa Fluor 647 from raw localization data and **e** with single-molecule fluorescence events combined into aggregates of ~2100 photons. **f** BetaII spectrin in the axon of a hippocampal neuron from rat. **g** PMP70 in peroxisomes in a Vero cell. A confocal scan of adjacently found peroxisomes is included for comparison. Please note that the images shown in (**e**, **f**) were derived from samples created by indirect immunofluorescence. Explanatory drawings adapted from ref. ^34^ (**a**) and ref. ^15^ (**f**). Source data are provided as a Source Data file. Scale bars: 50 nm (**a**), 2 nm (**c**), 250 nm (**d**), 100 nm (**e**), 200 nm (**f**, **g**).

To illustrate the performance of the instrument and benchmark against recent initial results^14^, we imaged Nup96, a nuclear pore complex (NPC) protein, labeled with the organic fluorophore Alexa Fluor 647 using SNAP-tag in fixed mammalian cells (Fig. 3a–e). Images of several square micrometers in size were acquired using MINFLUX sequences with TCP diameters and photon limits ranging from *L*_*p*_ = 300 nm to *L*_*f*_ = 40 nm and *N*_*p*_ = 40 to *N*_*f*_ = 350 (Fig. 2b–d). The example shown in Fig. 3e was acquired within ~10 min. The overall effective fluorescence detection rate of typically ~30 kHz allowed a complete localization within ~30 ms. To compensate for the reduction in excitation intensity with increasing match between the central donut zero and the fluorophore, and to keep up the fluorescence rate, we increased the intensity with each TCP iteration. Concretely, our continuous-wave donut-shaped excitation beam carried between 6 and 31 µW of average power at the sample.

Note that the associated focal intensities are approximately three times lower than in a confocal setting applying similar average power, because the focal cross-section of a donut is approximately three times larger than that of a regularly focused (Gaussian) beam.

To prompt a single active fluorophore per activation area, 10–700 nW of 405-nm light was applied until a fluorescent molecule appeared. To avoid simultaneous activation of several fluorophores, we interrupted the activation laser upon first detection of a fluorescence signal above the background level. From here, it took the microscope 28 ms on average to provide the first valid final localization of a newly activated fluorophore. Altogether, the acquisition of the NPCs in Fig. 3d (e) required about 40 (10) min, resulting in about 19,400 (410) final localizations. Our experiments demonstrated that our MINFLUX system, which is based on a standard inverted microscope stand, resolved the symmetry of Nup96 in single NPCs (Fig. 3e), distributed along a ring of ~110 nm in diameter, in line with previous initial results^14^. The localizations typically formed eight clusters of roughly two to four localization subclusters, displayed as the sum of Gaussian distributions, one for each localization, revealing individual Nup96 proteins through their individual fluorescent markers^4^.

MINFLUX imaging of the well-characterized Nup96-SNAP cell line^23^ also allows drawing quantitative conclusions on the localization precision achieved. To this end, we calculated the standard deviation $\sigma$ in the *x* and *y* directions of the estimated emitter positions from localization traces with four and more localizations per fluorophore. Each localization was obtained from an independent, aggregated segment of ~2100 photons from consecutive final ($L_f$ = 40 nm) iteration steps (see “Methods”). The resulting distribution of $\sigma$ featured a median of 0.9 nm (histogram in Fig. 3b). For a 2D assessment of the underlying precision, the mean established position of an emission train was subtracted from all individually obtained localizations. A single plot that compiles these differences for many localizations (Fig. 3c) highlights the precision attained and exhibits $\sigma$ ~ 1 nm, in agreement with Fig. 3b. An analysis of the scaling of $\sigma$ with photon number *N* in the final MINFLUX iterations is provided in Supplementary Fig. S3, revealing the known inverse square root relation. Supplementary Fig. S4 further explores this precision scaling and the nanoscope’s present technical precision limit of ~0.2–0.3 nm for *N* = 10^5^ photons at *L* = 40 nm (Supplementary Note 1).

Notably, although it is advantageous to use samples for MINFLUX which feature a short distance between fluorophore and structure of interest, we were able to acquire images with ~3-nm (standard deviation) localization precision (Supplementary Fig. S3), and consequently resolution of individually active fluorophores using classical indirect immunofluorescence labeling. We applied MINFLUX to immunolabeled spectrin residing in the highly periodic actin–spectrin protein lattice of a rat axon (Fig. 3f) and we visualized the protein PMP70 on the surface of peroxisomes (Fig. 3g) in Vero cells.

### Tracking biomolecules with microsecond-range time resolution with small fluorescent probes

**Fig. 4:**
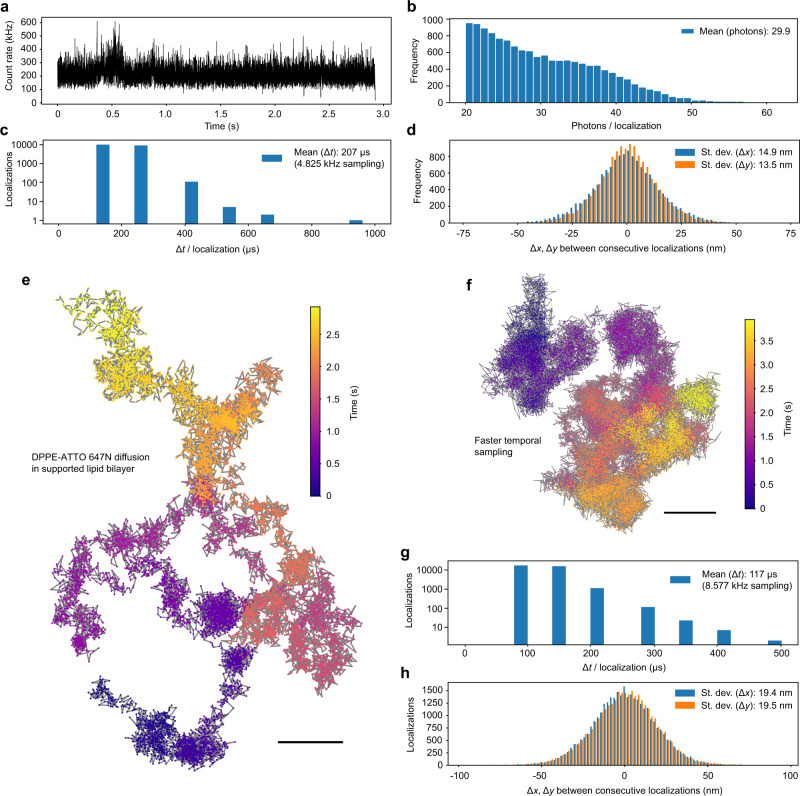
MINFLUX single-molecule tracking at ~100 µs temporal sampling. **a** Fluorescence intensity over time for a ~3-s excerpt of tracking DPPE-ATTO 647N lipid diffusion (at room temperature) in a supported lipid bilayer. **b** Histogram of photons per localization for the data shown in (**a**). **c** Duration of individual localizations. **d** Histogram of the standard deviations in the lateral displacements Δ*x* and Δ*y* of consecutive localizations, providing a conservative measure on the precision of individual localizations. **e** *xy* trajectory color coded to indicate the passage of time. **f** Enhanced temporal acquisition, sampling at 8.577 kHz (mean of 117 µs per localization). **g**, **h** Data as in (**c**, **d**) for the faster single-lipid tracking experiment. Source data are provided as a Source Data file. Scale bars: 200 nm (**e**, **f**).

We further illustrate the capabilities of MINFLUX for fast localization by tracking fluorescent lipid analogs in a defect-free supported lipid bilayer prepared on a coverglass, serving as a model membrane for lateral diffusion^4,24^. Lipids exhibit a high 2D diffusivity, and characterizing details of their diffusion in various biophysical contexts, including the plasma membrane of living cells^24–29^, is an important pursuit. We demonstrate that the diffusion of a 1,2-Dipalmitoyl-sn-glycero-3-phosphoethanolamine (DPPE) lipid labeled with the lipophilic dye ATTO 647N can be followed over extended spatial regions at time steps well below those accessible by popular camera tracking (Fig. 4). Operating at fluorescence count rates of 200 kHz (Fig. 4a) and ~30 photons per localization on average (Fig. 4b), MINFLUX tracking measured this diffusion at effective time steps down to Δ*t* ≈ 80.5 µs (Fig. 4c, g). Assessments of the lateral displacements Δ*x* and Δ*y* of consecutive localizations provide a conservative upper bound on the precision of individual localizations, which is <20 nm for localization frequencies up to 8.5 kHz (Δ*t*_mean_ ≈ 117 µs) effective position sampling (Fig. 4d, h).

The long trajectories (Supplementary Fig. S6) illustrate the capability to follow the diffusing lipid and reveal its intricate movement in high detail (Fig. 4e), making statistical evaluations more robust due to finer sampling. A slightly modified MINFLUX protocol in which the final-step TCP consists of three points rather than a hexagonal pattern additionally reduced the time required per localization (Fig. 2d). The result is a single-fluorophore tracking procedure that almost breaks the 100-µs mark of time resolution in the observation of single-fluorophore motion (Fig. 4f).

Due to the limited fluorescence rate afforded by small organic fluorophores, the high diffusivity exhibited by diffusing lipids was previously only just about within reach for a specialized confocalized tracking experiment that operated at the photon detection limit, albeit only over substantially smaller spatial domains^24^. Camera-based tracking remains substantially slower, averaging over at least a millisecond.

A spatiotemporal tracking method based on sequential position determination inevitably coarse grains the measured mobility of the moving molecule. This blurring effect arises from temporal averaging of position during the time interval elapsing while a localization is obtained. Signatures of hindered diffusion on small length scales, such as hop diffusion within nanoscale compartments or hierarchical interactions among lipids, proteins and cholesterol (CHOL), or any other transient effects, require very high temporal sampling for their visualization. This is why most studies of lipid diffusion have so far relied on labeling the lipids with strongly scattering but bulky gold particles of several tens of nanometers in size^27^. While the chemical nature of the fluorophore tag in the fluorescent lipid analog and potential effects of its interactions with other molecules remain highly relevant, an organic label that is at least ten times more compact than the gold particle is clearly more adequate. Besides, organic fluorophores seamlessly integrate into the membrane as a replacement of the lipid acyl chain. Owing to its strongly relaxed requirements on collected photon numbers required for nanometer-range localization, fluorophore tracking by MINFLUX should greatly propel the understanding of the diffusion of lipids and proteins in the cell membrane.

### 3D MINFLUX fluorescence imaging in cells

**Fig. 5:**
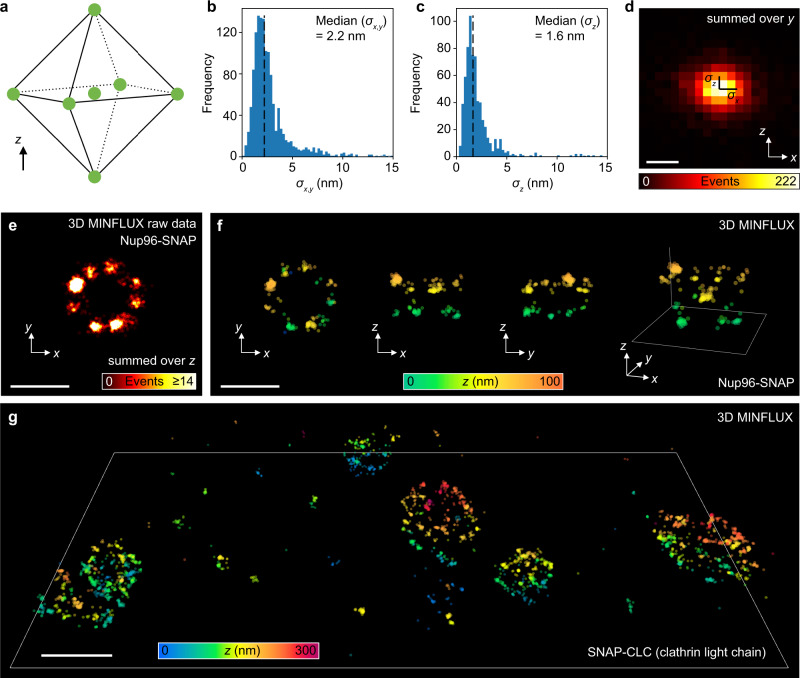
3D MINFLUX imaging. **a** Individual TCPs for iterative 3D MINFLUX are constructed from an octahedron-shaped coordinate set. Coordinates that deviate from the nominal focal plane are addressed by defocusing via a deformable mirror. **b** Lateral and **c** axial precision of Nup96-SNAP localizations, inferred from single-molecule fluorescence event aggregates of ~1200 photons. **d** Histogram of the distance between individual localizations of a single fluorophore and its mean position estimate. **e** Example of raw data and **f** rendering of a pore compare Supplementary Movie S1 for a large field of view. Panels (**b**–**d**) display pooled data from these data sets. **g** Clathrin visualized by SNAP labeling of the clathrin light chain in HeLa cells. The region demarcated by the white lines has an extent of 2.2 × 1.1 µm^2^. All SNAP-tags were labeled with Alexa Fluor 647. Source data are provided as a Source Data file. Scale bars: 4 nm (**d**), 100 nm (**e**, **f**), 200 nm (**g**).

Finally, we extended our MINFLUX imaging scheme to three spatial dimensions, following a similar approach as described in ref. ^14^. We programmed the SLM to shape the excitation focal intensity distribution into a 3D donut (also known as “bottle beam”), with a singular point of nearly zero intensity at its center, and modified the acquisition logic to scan TCPs that were constructed from the vertices of an upright octahedron (two opposing vertices each per *x*-, *y-*, and *z*-axis) and its center (Fig. 5a). To rapidly (~1 kHz) target out-of-plane (z ≠ 0) coordinates, we applied a variable amount of defocus to the DM in the common beam path of the microscope (Fig. 1). The obtained 3D images of SNAP-labeled Nup96 and Clathrin samples (Fig. 5e–g and Supplementary Movie S1) illustrate the 3D imaging performance of our setup. Based on the data obtained from aggregation of ~1200 photons from final iterations (*L*_*f*_ = 40 nm, *N*_*f*_ = 150), cluster analyses of these data sets as in the previous 2D experiment suggest a 3D localization precision of ~2.4 nm in the focal plane and ~1.9 nm along the optic axis (Fig. 5 and Supplementary Fig. S5).

As emphasized in this context before^14^, advanced data interpretation efforts will need to consider that fluorescence microscopes map (nothing but) the fluorophores as the observed molecules; they cannot map the targeted biomolecules by definition. In the case of MINFLUX nanoscopy, the fluorophore distribution is imaged with nanometer (3D) precision. Depending on the labeling strategy employed, displacements of the fluorophores from the actual target proteins can be substantial, in fact >10 nm in the case of indirect immunofluorescence labeling. Therefore, at this resolution, an image showing the fluorophore distribution cannot be readily equated with an image showing the respective distribution of the target biomolecule. The resolution obtained on the biomolecules inevitably depends on the fidelity and completeness^30^ of the biomolecular labeling.

Another factor to be observed is that fluorophores closer than 10 nm, i.e. within the FRET range, may increasingly show collective behavior. In particular, if their independent on/off switching is compromised, so is the ability of this concept to separate individual fluorophores. Further research of the fluorophore behavior at these distances is required. In any case, with the fluorophore resolution having reached fundamental limits, the restrictions set by labeling and the fluorophores themselves become increasingly relevant when drawing conclusions on the biomolecules at sub-10-nm length scales.

In the coming years, we expect a number of studies based on the application of MINFLUX to least-invasive small-molecule fluorescent tags. Our expectation is also based on the fact that, within <5 min after placing the sample on the microscope stage, our MINFLUX setup localized individual fluorophores with a standard deviation of ~1 nm, and ~2 nm in 3D. Thus true single digit nanometer far-field fluorescence imaging resolution and localization precision has become practicable. This and related instruments may therefore serve as a powerful and truly nanoscale imaging platform that is readily amenable to a wide range of applications in the life sciences.

## Methods

### Image rendering

Localization images are commonly rendered as a convolution of a point map of localizations with a Gaussian function. If the dynamic range becomes high, these images are often difficult to interpret as blooming from densely populated regions introduced by the tail of the Gaussian function obscures sparse events in the neighborhood. We therefore replaced the Gaussian with a two-dimensional top-hat-function of unity volume that, due to its limited support, does not induce blooming artifacts, and rendered MINFLUX raw data (Figs. 3d and 5e) and data from immunolabeled samples (Fig. 3f, g) accordingly. 3D localizations (Fig. 5) were rendered likewise as plain circles with diameter 3 nm and opacity 0.4 in Paraview^31^. The 2D data from the SNAP-labeled sample in Fig. 3e were rendered as described^14^, following the above-mentioned Gaussian approach using a sigma of 2 nm and a color map with a nonlinear brightness progression.

### Experimental setup

#### Fluorescence microscope

The optical setup (Fig. 1a, Supplementary Figs. S1, S4, S7, and Supplementary Notes 1 and 2) is based on a common fluorescence microscope (IX83, Olympus) that provides routinely needed features such as compatibility with standard stages (U-780.DOS, Physik Instrumente), sample holders (P-545.SH4, Physik Instrumente), brightfield and epifluorescence illumination (CoolLED pE-4000, CoolLED) with filter sets and eyepieces for quickly checking large ROIs in the sample. A 642 nm cw laser (2RU-VFL-P-1000-642-B1R, MPB Communications Inc.) was installed and focused in the focal plane of the ×100 magnification NA 1.4 oil objective lens (UPLSAPO100XO/1.4, Olympus). Two EODs (311A-AD*P/2, Conoptics) were installed in series with crossed (90°) deflection planes to enable fast lateral beam steering for MINFLUX measurements. A λ/2-plate placed in between the EODs rotates the laser beam polarization accordingly. A SLM (LCOS SLM X13268-13-01, Hamamatsu Photonics) controls the focal intensity distribution of the excitation light, which is subsequently superimposed with the detection beam path using a custom-made interference filter. A mirror-based beam scanning assembly consisting of a DM (Multi-C-1.5, Boston Micromachines Corp.) and a galvanometer scanner (QUAD Scanner) at the camera port of the microscope body steers the light to the sample, addressing a field of view up to 80 × 80 µm (for a ×100 objective). Supplementary Fig. S7 and Supplementary Note 2 detail the calibration of the EODs with respect to the galvanometer scanner. For activation of single fluorophores, we used a 405 nm wavelength laser (Cobolt MLD 405 nm 50 mW, HÜBNER Photonics) whose intensity was attenuated into the nW region by a neutral-density filter. After passing through a polarization-maintaining fiber, the activation laser was superimposed with the detection and the excitation beam path using a long-pass filter (ZET405NF, Chroma Technology). Fluorescence light emitted by the sample was collected by the objective lens, descanned by the scanning assembly, transmitted through the aforementioned filters, and passed to a confocal detection unit where fluorescence photons were counted by two avalanche photodiodes (SPCM-AQRH-13, Excelitas Technologies) detecting different spectral ranges. Data were collected using Abberior Instruments Imspector software (version 16) with MINFLUX drivers.

#### Stabilization unit

The stabilization beam path was largely distinct from the imaging arrangement (Fig. 1b and Supplementary Fig. S1). Light from a linearly polarized 980 nm cw laser (LP980-SF15, Thorlabs Inc.) was reflected by a polarizing beam splitter that separated the excitation from the detection path of the stabilization unit. Following a λ/4-plate for circular polarization, the laser beam was lifted by a periscope and passed a beam combiner that facilitated epifluorescence illumination. The beam entered the microscope stand (IX83 Two-deck System, Olympus) via the back port of the upper microscope deck where it was superimposed with the excitation beam path by reflecting of a short-pass filter (ZT1064 RDC-SP, Chroma Technology). The 980 nm laser was focused into the back aperture of the objective lens to illuminate the focal plane homogeneously. The light reflected by the sample (e.g., by the Au nanorods) took the same path back, reentering the stabilization unit. After passing the λ/4-plate for polarization transformation, the reflected light was transmitted by the PBS, filtered by an amplitude filter in a plane conjugated to the objective BFP, and focused onto a camera (DMK 33UX178, The Imaging Source) where it formed a widefield reflection image of the sample. To increase the signal-to-noise ratio, we suppressed light of wavelengths < 980 nm by placing a long-pass filter (808 LP Edge Basic, Semrock Inc.) in front of the camera. The sample was mounted on a three-axis piezo stage (P-545.3D8H, Physik Instrumente) that facilitated live sample realignment with respect to the objective lens.

#### Acquisition of localization traces and event filtering

Starting from the coordinates of the last successful localization or from a node of the search grid, the localization of a single emitter proceeds through a sequence of MINFLUX iteration steps. The position estimate obtained from a successful prior iteration is registered as the emitter coordinate, and a new localization round is initiated at this position. This new round involves the (re)centering of the EOD deflection field on this coordinate by the galvanometer scanner. As the emitter position is known at this point, the starting iteration is usually set to a late iteration step to conserve photons for subsequent localizations. A trace of localizations acquired in this manner stops as soon as one of two abort conditions comes into effect: (1) the emitter enters a dark state and does not reemit until a dark time of 3 ms is exceeded; (2) an iteration has been configured to include a probe position *p*_*o*_ at the TCP origin and a CFR test (related to the central donut ratio^12^), which then failed. For probe positions *p*_*j*_ with individual dwell times *t*_*j*_, the CFR:

1 $${\mathrm{CFR}}\left( {p_j} \right): = \frac{{p_0 \times \mathop {\sum }\nolimits_{j = 1}^m t_j}}{{t_0 \times \mathop {\sum }\nolimits_{j = 1}^m p_j}}$$

should stay <1 for a single emitter. If the CFR exceeds a set limit repeatedly (i.e., not due to transient background), the localization process abandons the last known emitter position.

A valid measure of the localization precision of a single immobile emitter is the spread of the estimated coordinates derived from its emission trace^14^. Likewise, individual trace entries can be aggregated into localizations with a higher number of contributing photons and thereby increased precision. We aggregated up to 6 (Fig. 3b, c, e and Supplementary Fig. S1) or 12 (Fig. 5) consecutive trace entries into final localizations, while allowing the last aggregate of a trace to comprise a lower number of entries. For moving emitters, a localization trace corresponds to a particle track. Tracking and imaging operation is therefore very similar, using sequences that are optimized for either speed or precision. While we did not further explore this option, this scheme lends itself to a combined imaging/tracking experiment during the same measurement.

By tuning the power of the activation light that is applied to the sample, the condition of one emitter in a bright state within the TCP can usually be satisfied with regard to separated emitters. However, fluorophores in closer proximity to each other (<about 6 nm) tend to interact and switch on together, which increases the probability of localizing their (emission weighted) mean instead of their individual positions. Transient background also has the potential to shift apparent emitter coordinates away from their true position in the sample. In practice, these effects contribute to a hazy background of localizations that can be reduced by subjecting the localization data to a cut with respect to the detected fluorescence rate and CFR. We applied upper limits for the effective fluorescence rate and CFR that has been detected during the final iteration of 50 kHz and no limit, or 200 kHz and 0.5, respectively, to the 2D data from Nup96-SNAP (Fig. 3d) and immunolabeled (Fig. 3f, g) samples.

### Sample preparation

#### Cell culture and immunolabeling

Mammalian cells were cultured in DMEM (Gibco) + GlutaMAX (Thermo Fisher Scientific) supplemented with 10% FBS (FBS Superior; Sigma-Aldrich), 1 mM sodium pyruvate (Thermo Fisher Scientific), and Penicillin-Streptomycin (100 µl/ml and 0.1 mg/ml; Sigma-Aldrich) at 37 °C and 5% CO_2_. Peroxisomes in Vero cells were labeled as described^4^. In brief, cells were fixed in 8% paraformaldehyde (PFA) in phosphate-buffered saline (PBS), permeabilized (0.5% Triton X-100/PBS), and blocked (2% BSA/0.1% Tween20/PBS). Then cells were incubated for 1 h using antibodies against PMP70 (1:200; abcam, code: ab3421). Primary antibodies were detected using secondary Alexa Fluor 647 AffiniPure Donkey Anti-Rabbit IgG (H + L) antibodies (1:400; Jackson ImmunoResearch, 711-605-152). Secondary antibodies were as well incubated for 1 h. Samples from neurons were prepared using primary antibodies against betaII spectrin (BD Biosciences, 612563) as described^32^. In brief, cultures of hippocampal neurons were prepared` from Wistar rats of mixed sex at the 1st postnatal day. After 20–30 days of cultivation on coated coverslips, cells were washed in PBS and fixed (4% PFA/PBS). After quenching the PFA (100 mM NH_4_Cl and 100 mM Glycine), permeabilization (0.1 % Triton X-100), and blocking (1% BSA/PBS), primary and secondary antibody incubations were performed (in PBS) for 1 h at room temperature.

#### SNAP labeling

NUP samples were prepared as described previously^23^. In brief, U2OS cell stably expressing Nup96-SNAP (ATCC-HTB-96, Lot # 61074667) were seeded on coverslips. After a prefixation (2.4% PFA; 30 s) and permeabilization (0.4% Triton X-100; 3 min), cells were fixed in 2.4% PFA for 30 min. After quenching the PFA (50 mM NH_4_Cl) and washing (PBS; 2x 5 min), blocking was performed with ImageIT Signal Enhancer (Thermo Fisher) for 30 min. For staining, cells were incubated with 1 µM SNAP-Surface Alexa Fluor 647 (Thermo Fisher) in PBS/0.5% BSA/1 mM DTT for 50 min. Before mounting, the samples were washed three times with PBS for 5 min. For imaging clathrin coated vesicles, HeLa cells were transfected with SNAP-CLC^33^ using Turbofect (Thermo Fisher) according to the manufacturers' protocol. Then the cells were labeled as described for Nup96-SNAP cells. The coverslips carrying the cells were prepared as described in the sample mounting and imaging buffers section.

#### Sample mounting and imaging buffers

For the sample stabilization during MINFLUX measurements, gold nanorods (A12-40-980-CTAB-DIH-1-25, Nanopartz Inc.) were used as fiducials as described before^12^. In brief, an undiluted dispersion of the nanorods was applied to the ready-made samples. Before mounting the samples in imaging buffer, the coverslips were rinsed several times with phosphate-buffered saline (PBS) to remove unbound nanorods. For MINFLUX imaging of samples labeled with Alexa Fluor 647, GLOX buffer (50 mM TRIS/HCl, 10 mM NaCl, 10% (w/v) Glucose, 64 µg/ml catalase, 0.4 mg/ml glucose oxidase, 10–25 mM MEA, pH 8.0) was used^12,14^. Samples were sealed with twinsil (picodent).

#### Supported lipid bilayers

For tracking, a solution consisting of 1,2-dioleoyl-sn-glycero-3-phosphocholine (*Avanti*): CHOL (*Avanti*) and Abberior STAR 488-CHOL (50:49.5:0.5), doped with ATTO 647N-DPPE (*ATTO-TEC GmbH*) was dissolved in 50/50 chloroform/ethanol. Supported lipid bilayers were created by spin coating (3000 rpm for 30 s) this solution onto coverslips. To keep the layer hydrated, the coverslip was placed in a sample chamber and covered with PBS.

### Statistics and reproducibility

All experiments were repeated three or more times with similar results.

### Reporting summary

Further information on research design is available in the Nature Research Reporting Summary linked to this article.

## Supplementary information

Supplementary Information

Supplementary Software

Supplementary Movie 1

Reporting Summary

Description of Additional Supplementary Files

## Data Availability

Source data are provided with this paper. The authors declare that all other data supporting the findings of this study are available within the paper and its Supplementary information files.

## Source data

Source Data

## Code availability

A software package of custom-written Python scripts and illustrative data sets is included in the Supplementary Software file.
