# Supplementary material for: MINFLUX nanometer-scale 3D imaging and microsecond-range tracking on a common fluorescence microscope
Source: Nat Commun. 2021 Mar 5;12:1478. doi: 10.1038/s41467-021-21652-z (PMC7935904; doi:10.1038/s41467-021-21652-z)
Supplement: Supplementary file 1 — Supplementary Information [file 41467_2021_21652_MOESM1_ESM.pdf]

Supplementary Information for

MINFLUX nanometer-scale 3D imaging and microsecond-range tracking on a common fluorescence microscope

Roman Schmidt[1*], Tobias Weihs[1], Christian A. Wurm[1,2], Isabelle Jansen[1], Jasmin Rehman[2], Steffen J. Sahl[3] and Stefan W. Hell[3,4,*]

[1]Abberior Instruments GmbH, Göttingen, Germany; [2]Abberior GmbH, Göttingen, Germany
[3]Max Planck Institute for Biophysical Chemistry, Department of NanoBiophotonics, Göttingen, Germany
[4]Max Planck Institute for Medical Research, Department of Optical Nanoscopy, Heidelberg, Germany

* Email: r.schmidt@abberior-instruments.com and stefan.hell@mpibpc.mpg.de.

Contents

Page

2 Supplementary Fig. S1. Overview of the stabilization unit.

3 Supplementary Fig. S2. Impact of TCP shape on estimator performance.

4 Supplementary Fig. S3. Photon dependence of the localization precision.

5 Supplementary Fig. S4. Microscope lower precision limit measured with a technical 40 nm bead sample.

5 Supplementary Note 1. Microscope lower precision limit.

6 Supplementary Fig. S5. Localization precision in 3D imaging experiment of clathrin in HeLa cell.

7 Supplementary Fig. S6. Distribution of track durations for single lipid tracking.

8 Supplementary Note 2. Calibration of the tandem scanner.

9 Supplementary Fig. S7. Calibration of the tandem scanner.

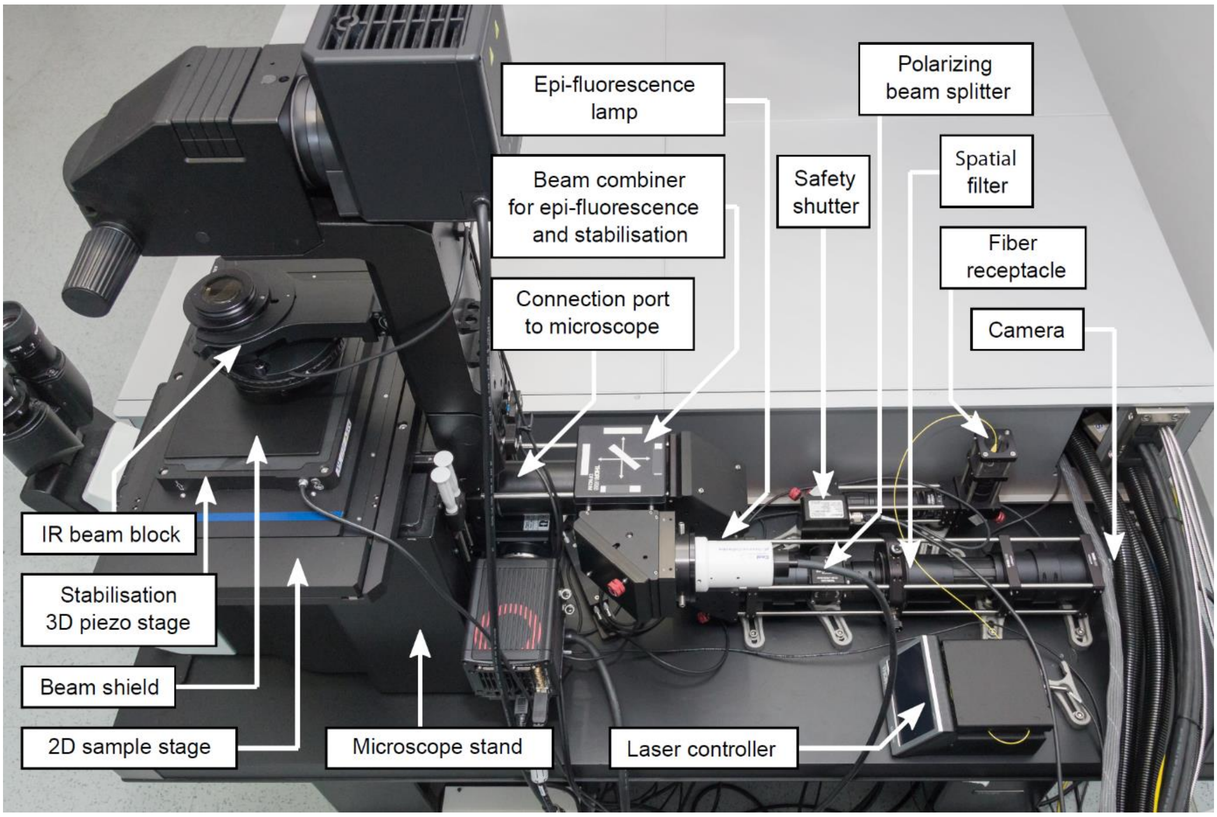

Supplementary Fig. S1. Overview of the stabilization unit. The epi-fluorescence beam path and laser safety equipment (fast safety shutter, IR beam block and beam shield) are visible along the excitation and detection beam path of the stabilization unit. A tight cable routing blocks the view of the stabilization camera.

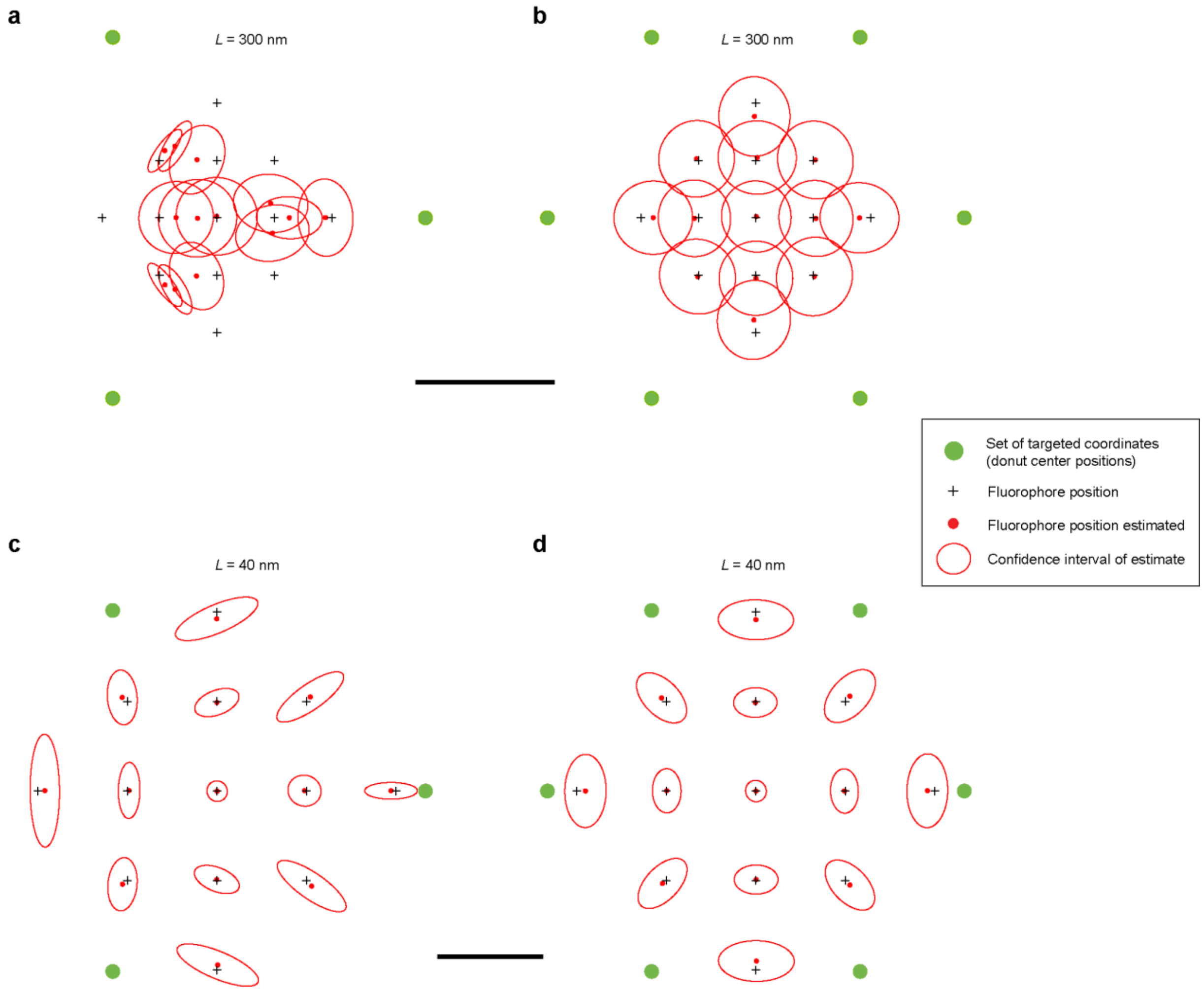

Supplementary Fig. S2. Impact of TCP shape on estimator performance. Simulated molecules at positions (crosses) within the FOV and the distributions (red) of their respective estimate represented by their mean (dot) and confidence intervals (ellipsoids) for triangular (a, c) and hexagonal (b, d) TCPs with diameters *L* of 300 nm (a,b) and 40 nm (c,d). Real-time unbiasing was applied to the radial coordinate only. The benefit of the improved angular uniformity of the localization precision (i.e., low anisotropy in estimator bias) of a hexagonal TCP becomes particularly noteworthy for emitters that reside at the periphery of a TCP with a large diameter. Source data are provided as a Source Data file. Scale bars: 100 nm (a,b), 10 nm (c,d).

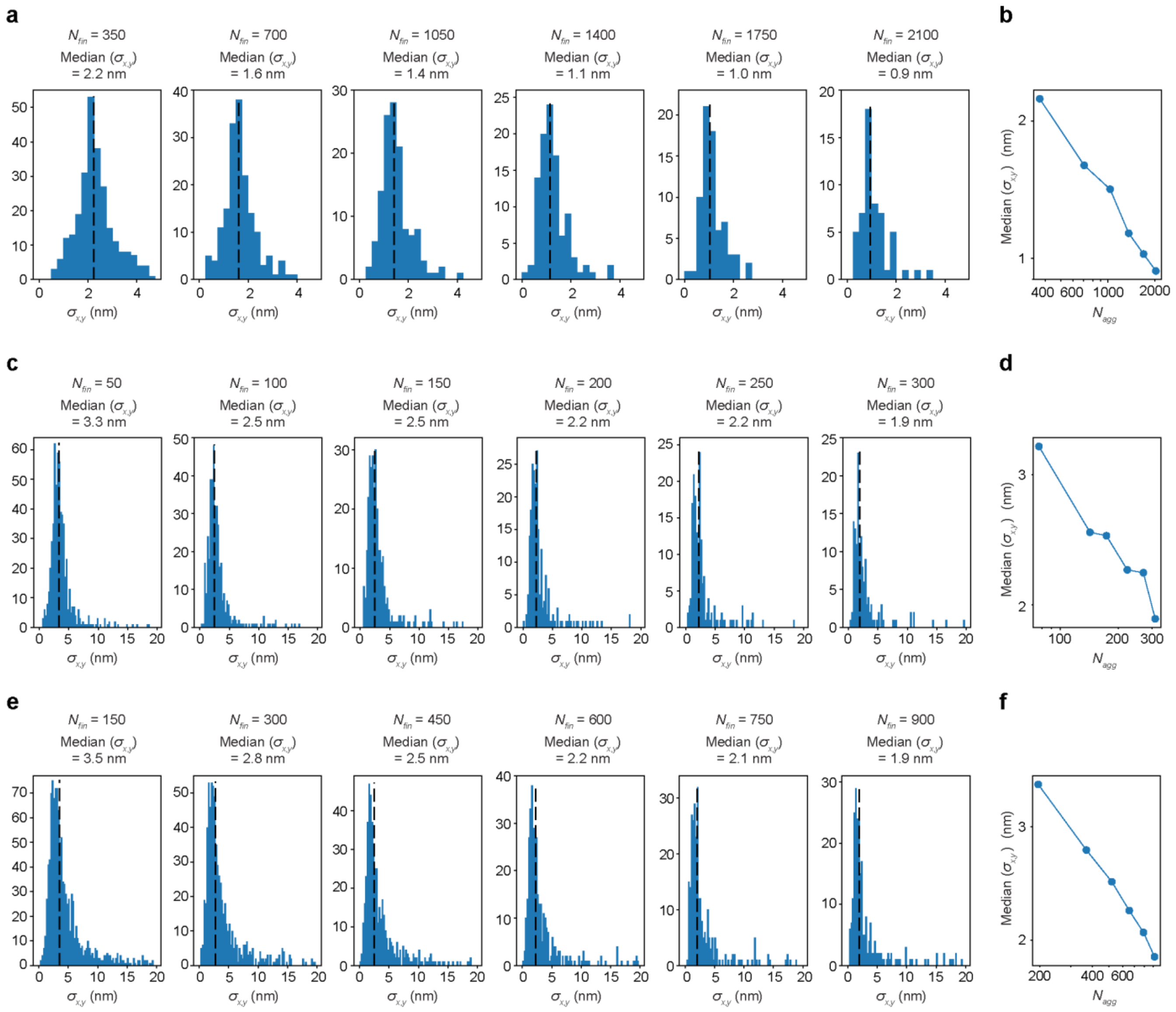

Supplementary Fig. S3. Photon dependence of the localization precision. Standard deviations of groups of ≥4 successive localizations obtained from single-fluorophore photon emission bursts establish the localization precision (data from the Nup96 recording shown in Fig. 3d). The localizations are derived from successive and separate groups of N_{fin} photons from each burst, from the final MINFLUX iterations. **a**, Histograms of $\sigma_{x,y}$ for different photon numbers N_{fin}. **b**, Median of $\sigma_{x,y}$ vs. mean of photons per aggregate N_{agg} (log-log representation). **c,d** Data as in a,b for the spectrin recording of Fig. 3f. **e,f** Data as in a,b for the PMP70 recording of Fig. 3g. Source data are provided as a Source Data file.

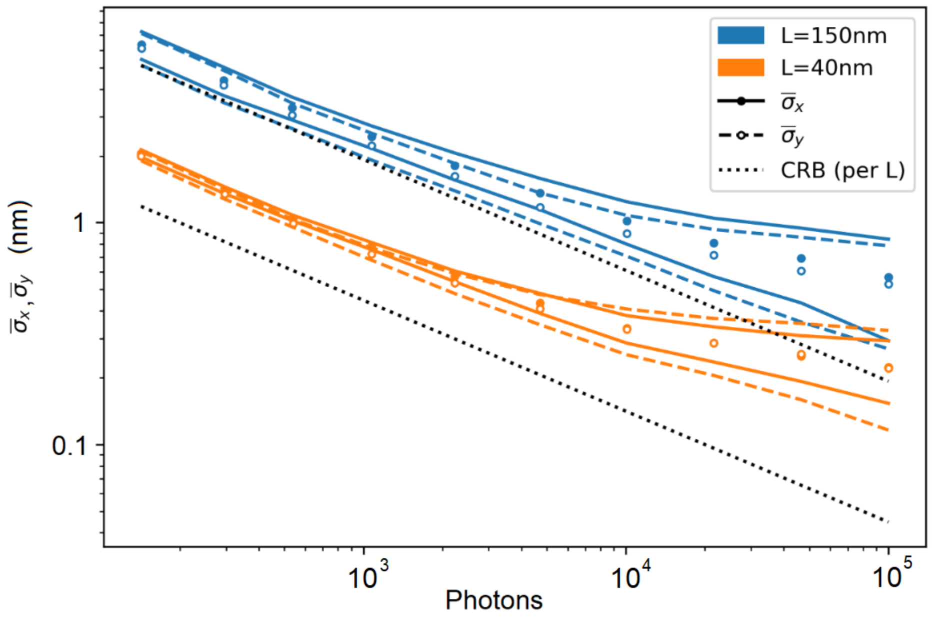

Supplementary Fig. S4. Microscope precision limit measured with a 40 nm bead sample. Plotted is the mean standard variation of bead localizations along the *x* and *y* direction and their respective one-sigma confidence intervals (bounded by solid and dashed lines for x- and y direction, respectively), versus the number of photons used per localization for pattern diameters *L* of 150 nm and 40 nm. Source data are provided as a Source Data file.

Supplementary Note 1. Microscope precision limit.

The theoretical precision limit given by the Cramér-Rao lower bound (CRLB) scales down to arbitrarily small values in proportion to 1/sqrt(*N*), with N denoting the photons detected per localization. The practical limit of the precision is finite though, as it is influenced by the positioning noise of the sample stabilization and the (galvanometer) beam steering. Therefore, in order to characterize the performance of the microscope we measured the spread of repeated localizations using a "good-natured" technical sample. Supplementary Fig. S4 summarizes the localization data that we obtained from optically stabilized (Fig. 1) measurements of 40 nm fluorescent beads at a photon detection rate of ~450 kHz. For varying *L* values, we recorded about $4{\cdot}10^6$ photons from an individual bead, distributed over >23 independent acquisitions. To estimate the localization error, we binned these photon traces into groups of about *N* sequential photons, estimated the bead position from each group and calculated the standard deviation of the positions that we obtained from each trace.

The plot of the mean sigma versus the number *N* of photons used per localization scales with the respective CRLB down to a precision of ~1 nm, after which the plots start to flatten out. This result indicates an effective noise floor and a limit for the obtainable precision. For $N = 10^5$ photons and L = 40 nm this precision limit is close to 0.2 nm.

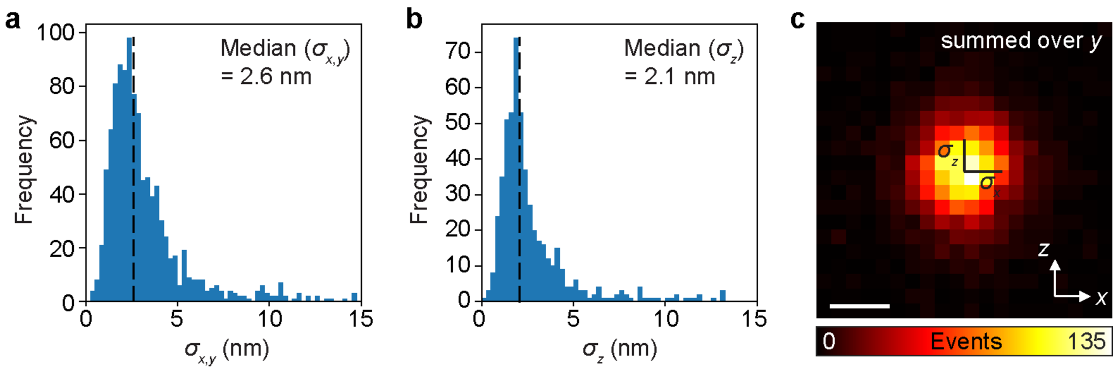

Supplementary Fig. S5. Localization precision in 3D imaging experiment of clathrin in HeLa cell. a, Lateral and **b**, axial precision of SNAP-CLC localizations, inferred from single-molecule fluorescence event aggregates of ~1200 photons. **c**, Histogram of the distance between individual localizations of a single fluorophore and its mean position estimate. Source data are provided as a Source Data file. Scale bar: 4 nm (c).

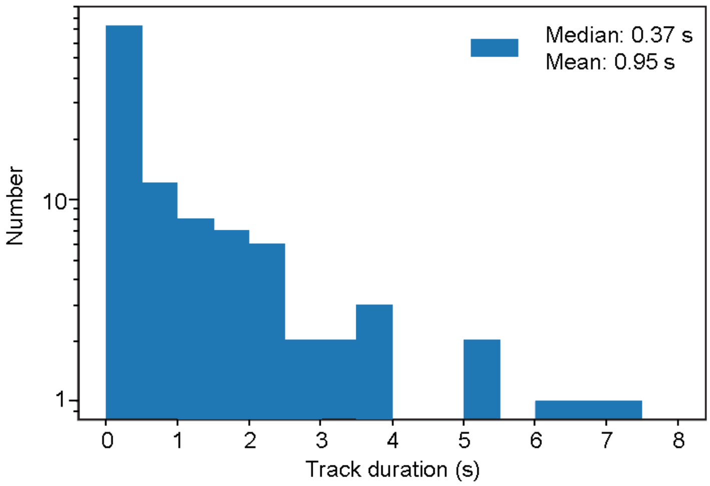

Supplementary Fig. S6. Distribution of track durations for single lipid tracking. Histogram of track durations of fluorophores coupled to lipids diffusing in a supported lipid bilayer acquired in MINFLUX tracking experiments using the enhanced temporal acquisition scheme (compare Fig. 4 f, g and h). The data shown is filtered for tracks with mean localization rates ≥6.67 kHz (≤150 µs in between consecutive localizations) and ≤250 kHz mean count rate. Source data are provided as a Source Data file.

Supplementary Note 2. Calibration of the tandem scanner.

The tandem scanner of the microscope steers the probe beam to a target position inside the sample by two crossed electro-optical deflectors (EODs) followed by a galvanometric quad-scanner. To address a specific position inside the focal plane, the coordinate frames of these two systems need to be registered with respect to each other. We solved this by recording four scanning images of the same fluorescent bead, with an open pinhole and with a different pair of voltages

$$\boldsymbol{V}_0^+, \boldsymbol{V}_0^-, \boldsymbol{V}_1^+, \boldsymbol{V}_1^- = (+125V, 0V), (-125V, 0V), (0V, +125V), (0V, -125V)$$

applied to both EODs during each scan, and fitted the resulting apparent bead positions $\boldsymbol{m}_{0,1}^{+,-}$(Supplementary Fig. S2). Writing $\boldsymbol{\delta m}_i := \boldsymbol{m}_i^+ - \boldsymbol{m}_i^-$ and $\boldsymbol{\delta V}_i := \boldsymbol{V}_i^+ - \boldsymbol{V}_i^-$ for the differences of measured position vectors and applied voltage vectors, we obtain the transformation matrix

$$\textbf{GrEV} = -1 \cdot \begin{bmatrix} \boldsymbol{\delta m}_0 \\ \boldsymbol{\delta m}_1 \end{bmatrix}^{-1} \cdot \begin{bmatrix} \boldsymbol{\delta V}_0 \\ \boldsymbol{\delta V}_1 \end{bmatrix}$$

between the intended beam displacement $\boldsymbol{r}$ and the necessary EOD voltages $\boldsymbol{V}$:

$$\begin{pmatrix} V_0 \\ V_1 \end{pmatrix} = \textbf{GrEV} \cdot \begin{pmatrix} r_0 \\ r_1 \end{pmatrix}$$

Note that any beam displacement by the EOD unit will shift the bead position in the scanning image (which is indexed by the galvo axis coordinates) to the opposite direction, and hence generates a sign change in the expression for GrEV above). Covering a larger grid area with more sampling points would additionally allow to measure and compensate non-linearities in the beam displacement generated by the EODs. In practice however, the linear approach sufficed, since the maximum voltages (±150 V) applied during our experiments are moderate compared to the ±500 V limit of the EODs used.

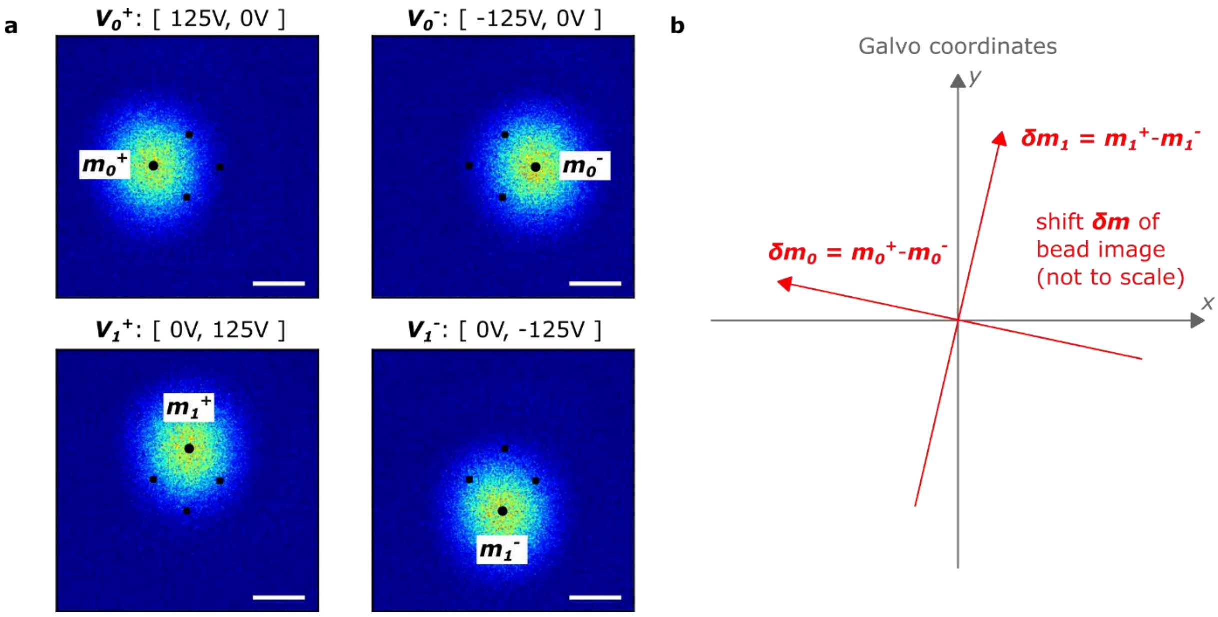

Supplementary Fig. S7. Calibration of the tandem scanner. a, Open-pinhole scans of a 20 nm fluorescent bead, taken at the same coordinates of the galvo scanner, but with different voltage pairs $\boldsymbol{V}$ applied to the axes of the electro-optical deflection unit. Black crosses mark the fitted apparent bead positions of all four recordings, from which **b** the transformation matrix GrEV, that connects a spatial beam displacement $\boldsymbol{r}$ in quad-scanner (resp. image) coordinates with the applied EOD voltages $\boldsymbol{V}$, was calculated (illustration not to scale). Scale bars 200 nm. Source data are provided as a Source Data file.
