# Supplementary figures and images for: MINFLUX nanometer-scale 3D imaging and microsecond-range tracking on a common fluorescence microscope
Source: Nat Commun. 2021 Mar 5;12:1478. doi: 10.1038/s41467-021-21652-z (PMC7935904; doi:10.1038/s41467-021-21652-z)

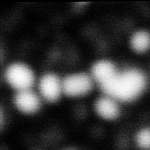

Supplement: Supplementary file 2 — Supplementary Software [file 41467_2021_21652_MOESM2_ESM.zip › PMP70_confocal_pixelsize20nm.png]

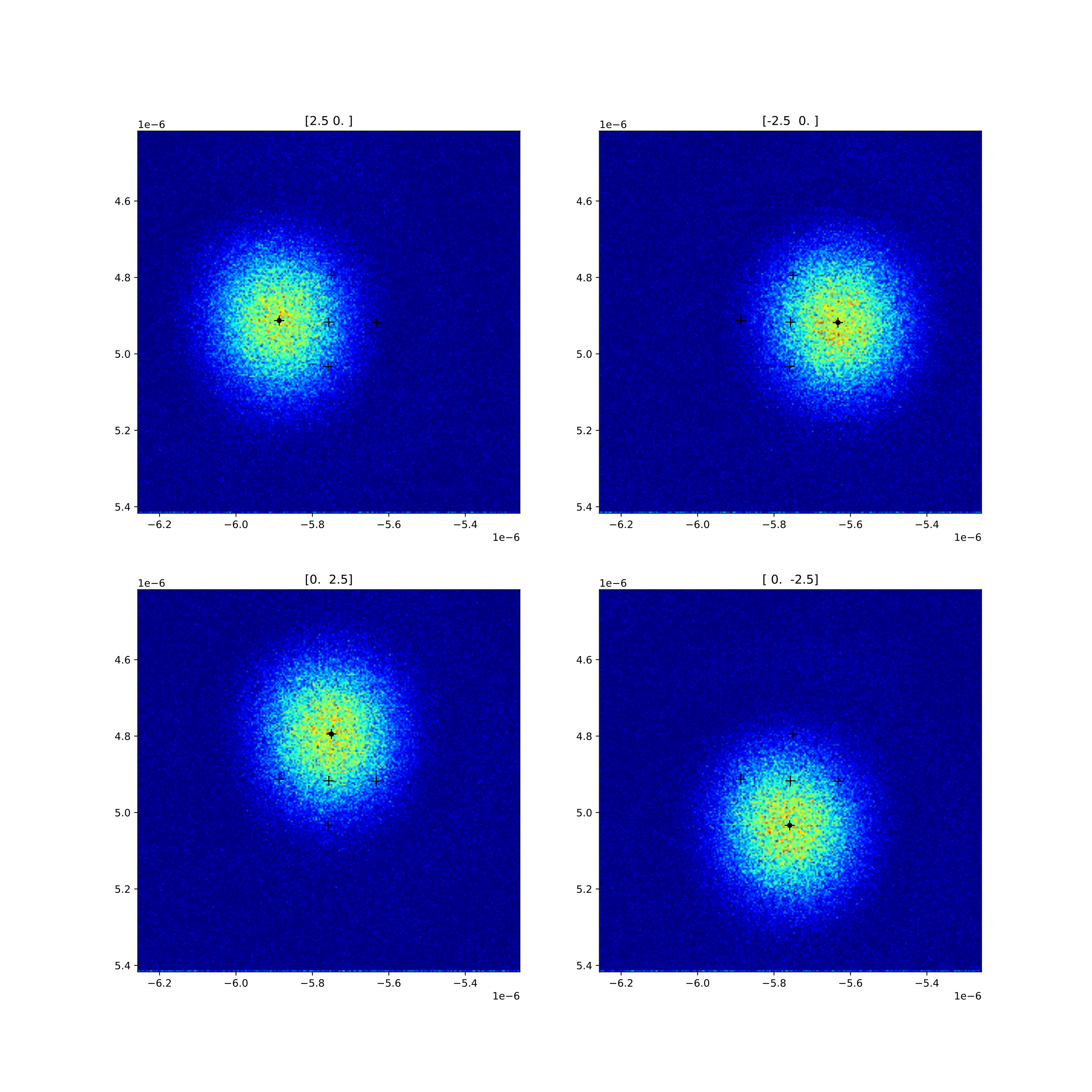

Supplement: Supplementary file 6 — Source Data [file 41467_2021_21652_MOESM6_ESM.zip › Figure_S7.png]
