# Supplementary material for: MINFLUX nanometer-scale 3D imaging and microsecond-range tracking on a common fluorescence microscope
Source: Nat Commun. 2021 Mar 5;12:1478. doi: 10.1038/s41467-021-21652-z (PMC7935904; doi:10.1038/s41467-021-21652-z)
Supplement: Supplementary file 4 — Reporting Summary [file 41467_2021_21652_MOESM4_ESM.pdf]

nature research

Corresponding author(s): Stefan W. Hell and Roman Schmidt

Last updated by author(s): Jan 28, 2021

Reporting Summary

Nature Research wishes to improve the reproducibility of the work that we publish. This form provides structure for consistency and transparency in reporting. For further information on Nature Research policies, see our Editorial Policies and the Editorial Policy Checklist.

Statistics

For all statistical analyses, confirm that the following items are present in the figure legend, table legend, main text, or Methods section.

n/a	Confirmed	
x		The exact sample size (*n*) for each experimental group/condition, given as a discrete number and unit of measurement
x		A statement on whether measurements were taken from distinct samples or whether the same sample was measured repeatedly
x		The statistical test(s) used AND whether they are one- or two-sided<br>*Only common tests should be described solely by name; describe more complex techniques in the Methods section.*
x		A description of all covariates tested
x		A description of any assumptions or corrections, such as tests of normality and adjustment for multiple comparisons
	x	A full description of the statistical parameters including central tendency (e.g. means) or other basic estimates (e.g. regression coefficient) AND variation (e.g. standard deviation) or associated estimates of uncertainty (e.g. confidence intervals)
x		For null hypothesis testing, the test statistic (e.g. *F*, *t*, *r*) with confidence intervals, effect sizes, degrees of freedom and *P* value noted<br>*Give P values as exact values whenever suitable.*
x		For Bayesian analysis, information on the choice of priors and Markov chain Monte Carlo settings
x		For hierarchical and complex designs, identification of the appropriate level for tests and full reporting of outcomes
x		Estimates of effect sizes (e.g. Cohen's *d*, Pearson's *r*), indicating how they were calculated

Our web collection on statistics for biologists contains articles on many of the points above.

Software and code

Policy information about availability of computer code

Data collection	Abberior Instruments Imspector software (version 16) with MINFLUX drivers
Data analysis	Custom written software in Python version 3.7.

For manuscripts utilizing custom algorithms or software that are central to the research but not yet described in published literature, software must be made available to editors and reviewers. We strongly encourage code deposition in a community repository (e.g. GitHub). See the Nature Research guidelines for submitting code & software for further information.

Data

Policy information about availability of data

All manuscripts must include a data availability statement. This statement should provide the following information, where applicable:

- Accession codes, unique identifiers, or web links for publicly available datasets
- A list of figures that have associated raw data
- A description of any restrictions on data availability

The data that support the findings of this study are available from the corresponding author S.W.H. upon reasonable request.

Field-specific reporting

Life sciences study design

All studies must disclose on these points even when the disclosure is negative.

Sample size	No sample-size calculation was performed. The manuscript reports the demonstration of an imaging method, but draws no biological conclusions, and does not examine or compare different biological conditions. This is not a life science study with comparative analyses of a certain sample size.
Data exclusions	No data was excluded from the analysis.
Replication	All attempts at replication were successful. All experiments were repeated three or more times with similar results.
Randomization	No randomization was performed. This is not a life science study with comparative analyses of biological situations.
Blinding	No blinding was performed. There is no comparison of different biological situations performed in this work.

Reporting for specific materials, systems and methods

We require information from authors about some types of materials, experimental systems and methods used in many studies. Here, indicate whether each material, system or method listed is relevant to your study. If you are not sure if a list item applies to your research, read the appropriate section before selecting a response.

Materials & experimental systems

n/a	Involved in the study	
☐	☒	Antibodies
☐	☒	Eukaryotic cell lines
☒	☐	Palaeontology and archaeology
☒	☐	Animals and other organisms
☒	☐	Human research participants
☒	☐	Clinical data
☒	☐	Dual use research of concern

Methods

n/a	Involved in the study	
☒	☐	ChIP-seq
☒	☐	Flow cytometry
☒	☐	MRI-based neuroimaging

Antibodies

Antibodies used	Anti-PMP70 antibody (1:200; abcam, catalog number ab3421); secondary Alexa Fluor® 647 AffiniPure Donkey Anti-Rabbit IgG (H+L) antibodies (1:400; Jackson ImmunoResearch, code: 711-605-152); anti-betaII spectrin antibody (BD Biosciences, catalog number 612563)
Validation	Commercially available antibodies were applied to create specimen for testing the described microscopy technique. Validations were performed by the manufacturers: Abcam ab3421 was tested by Western blot and immunofluorescence analysis in various cell lines (https://www.abcam.com/pmp70- antibody-ab3421.html); BD BioSciences 612563 routinely tested by Western blot (https://www.bdbiosciences.com/us/reagents/ research/antibodies-buffers/cell-biology-reagents/cell-biology-antibodies/purified-mouse-anti---spectrin-ii-42b-spectrin-ii/ p/612563).

Eukaryotic cell lines

Policy information about cell lines

Cell line source(s)	ATCC-HTB-96, Lot # 61074667. The cells can be obtained via Cell Line Services (CLS, clsgmbh.de, Nup96-SNAP #300444). VERO-B4 cells were obtained from the German Collection of Microorganisms and Cell Cultures GmbH (https://www.dsmz.de/ collection/catalogue/details/culture/ACC-33).
Authentication	Cell lines were not further authenticated.
Mycoplasma contamination	Cells were tested negative for mycoplasma contamination.
Commonly misidentified lines (See ICLAC register)	No commonly misidentified cell lines were used.
