# Supplementary material for: MINFLUX nanometer-scale 3D imaging and microsecond-range tracking on a common fluorescence microscope
Source: Nat Commun. 2021 Mar 5;12:1478. doi: 10.1038/s41467-021-21652-z (PMC7935904; doi:10.1038/s41467-021-21652-z)
Supplement: Supplementary file 5 — Description of Additional Supplementary Files [file 41467_2021_21652_MOESM5_ESM.pdf]

Title: Supplementary Movie 1

Description: 3D MINFLUX imaging of nuclear pore protein Nup96. Animated fly-through of the 3D dataset, colorcoded according to axial position z.

Title: Supplementary Software

Description:

Software (Python script) to generate MINFLUX images from instrument data. The example datasets are raw data (*.json) of measurements discussed in the manuscript. The expected output is the MINFLUX images (*.png) as shown in the manuscript.
